# The Royal Free Hospital's New Out-Patient Department

**Published:** 1912-11-09

**Authors:** 

**Affiliations:** Chairman of the Committee of Management, Royal Free Hospital, Gray's Inn Road, London, W.C.


*To the Editor of* The Hospital.

### The Royal Free Hospital's New Out-Patient Department.

*To the Editor of* The Hospital.

Sir,—I earnestly ask your aid in bringing before your readers an appeal on behalf of this hospital. A new out-patient department and certain other additions have been urgently needed for many years; the recent acquisition of about one acre of land has made the carrying out of these improvements possible.

The need of efficient accommodation at this hospital for the treatment of out-patients has been recognised by King Edward's Hospital Fund, and the Committee have determined to proceed with this extension at once. I therefore appeal to those who sympathise with the great work carried on at this, in common with other hospitals, for the benefit of the sick and suffering, to give their ready aid in supporting an institution which was the first to open its doors free to the necessitous poor, and to provide medical treatment without payment or letters of recommendation. The benefits of this hospital in ministering to the needs of the sick poor are well known, and as an institution providing facilities for the medical education of women, this part of its work is an additional claim to the support of many. By reason of these facilities the work of the hospital is more far-reaching and widely spread than is generally recognised, and, apart from its claim upon the benevolent for charitable support, financial aid is sought from those who recognise that advantages are gained by the whole country through the services of fully qualified medical women, able to fill positions suitable to their special abilities. The annual attendances of out-patients number over 106,000, and these are treated at present in quarters entirely unsuitable and ill-adapted for the purpose. The annual expenditure amounts to nearly twenty thousand pounds, only one-fifth of which is assured, the remainder having to be raised from voluntary sources or by bequests from friends.

The intention of the Committee to equip the Royal Free Hospital in accordance with modern requirements will necessitate the expenditure of about £50,000, and an urgent appeal is made for donations for raising this sum. Donations will be gratefully acknowledged by the Honorary Treasurers, and should be sent to Messrs. Lloyds Bank, Holborn Circus Branch, or to the Secretary, Royal Free Hospital, Gray's Inn Road, W.C.—Your obedient servant,

Sandwich,

Chairman of the Committee of Management.

Royal Free Hospital, Gray's Inn Road,
London, W.C., November 2.

